# The Commonly Used Bactericide Bismerthiazol Promotes Rice Defenses against Herbivores

**DOI:** 10.3390/ijms19051271

**Published:** 2018-04-24

**Authors:** Pengyong Zhou, Xiaochang Mo, Wanwan Wang, Xia Chen, Yonggen Lou

**Affiliations:** State Key Laboratory of Rice Biology & Ministry of Agriculture Key Lab of Molecular Biology of Crop Pathogens and Insects, Institute of Insect Sciences, Zhejiang University, Hangzhou 310058, China; zhoupy1991@163.com (P.Z.); camelotmo@zju.edu.cn (X.M.); wan456wan@126.com (W.W.); 21317040@zju.edu.cn (X.C.)

**Keywords:** bismerthiazol, rice, induced defense responses, chemical elicitors, *Sogatella furcifera*, defense-related signaling pathways

## Abstract

Chemical elicitors that enhance plant resistance to pathogens have been extensively studied, however, chemical elicitors that induce plant defenses against insect pests have received little attention. Here, we found that the exogenous application of a commonly used bactericide, bismerthiazol, on rice induced the biosynthesis of constitutive and/or elicited jasmonic acid (JA), jasmonoyl-isoleucine conjugate (JA-Ile), ethylene and H_2_O_2_ but not salicylic acid. These activated signaling pathways altered the volatile profile of rice plants. White-backed planthopper (WBPH, *Sogatella furcifera*) nymphs and gravid females showed a preference for feeding and/or oviposition on control plants: survival rates were better and more eggs were laid than on bismerthiazol-treated plants. Moreover, bismerthiazol treatment also increased both the parasitism rate of WBPH eggs laid on plants in the field by *Anagrus nilaparvatae*, and also the resistance of rice to the brown planthopper (BPH) *Nilaparvata lugens* and the striped stem borer (SSB) *Chilo suppressalis*. These findings suggest that the bactericide bismerthiazol can induce the direct and/or indirect resistance of rice to multiple insect pests, and so can be used as a broad-spectrum chemical elicitor.

## 1. Introduction

To protect themselves from damage by herbivores, plants have constitutive and induced defense systems. Constitutive defenses are defense traits that exist in plants whether or not herbivore infestation occurs. Induced defenses are defense traits that appear only when plants are exposed to herbivory [1,2]. Induced defenses in plants are activated by herbivore-associated signals and regulated by a complex signaling network, which mainly includes mitogen-activated protein kinase cascades and pathways mediated by jasmonic acid (JA), jasmonoyl-isoleucine (JA-Ile), salicylic acid (SA), ethylene (ET) and reactive oxygen species [3,4,5,6]. Regulation in plants leads to large changes in transcriptomes, proteomes and metabolomes; these changes may reduce the palatability of herbivores and increase their attractiveness to the natural enemies of herbivores, thereby increasing the direct and/or indirect resistance of plants to herbivores [3].

Induced plant defenses can also be triggered by applying chemicals that elicit plant resistance to herbivores or pathogens [7,8]. These chemicals are called chemical elicitors. Thus far, many potential chemical elicitors that induce the resistance of plants to pathogens have been reported, and several such elicitors—for example, benzo (1,2,3) thiadiazole-7-carbothioic acid *S*-methyl ester (BTH)—have been used commercially [9,10]. However, chemical elicitors that induce plant defenses against insect pests have received little attention. So far, JA and methyl ester of JA (MeJA) have been the most studied and are considered the most active chemical elicitors. They can activate diverse defensive compounds in plants, including polyphenol oxidase, proteinase inhibitors and volatiles [11,12,13,14], thereby leading to the direct and indirect resistance of plants to herbivores [1,15,16,17]. Recently, several JA analogues, such as coronalon, 6-ethyl indanoyl isoleucine conjugate, *cis*-jasmone JA-Ile and JA–amino acid conjugates, have been synthesized and found to induce herbivore resistance in plants [18,19,20,21]. Xin et al. [8] reported that the exogenous application of an herbicide, 2,4-dichlorophenoxyacetic acid (2,4-D), can induce the production of trypsin proteinase inhibitors (TrypPIs) and volatiles in rice, which in turn enhance the resistance of rice to the striped stem borer (SSB) *Chilosuppressalis* and the attractiveness of rice to brown planthopper (BPH) *Nilaparvata lugens* (Stål) and its egg parasitoid *Anagrus nilaparvatae*. In addition, several chemical elicitors, such as BTH, laminarin, tiadinil and *cis*-jasmone, have been reported to elicit indirect defenses in plants [22,23,24,25].

Bismerthiazol is a commonly used bactericide for the control of rice diseases caused by *Xanthomonas oryzae pv. Oryzae* (*Xoo*), such as bacterial rice leaf blight and bacterial leaf streak, and citrus canker caused by *X. campestris pv. Citri* [26,27,28]. In addition to having direct anti-microbial properties, bismerthiazol can also enhance H_2_O_2_ production, cellular defense responses and defense-related gene expression in *Xoo*-inoculated rice leaves [29].

Rice, one of the most important crops in the world, is infested by many insect pests, such as the white-backed planthopper (WBPH) *Sogatella furcifera*, BPH and SSB [30]. Previous studies with rice have shown that herbivore attack induces the biosynthesis of a variety of defense-related signals including JA, JA-Ile, SA, H_2_O_2_ and ET; these, in turn, regulate defense responses, such as the release of herbivore-induced volatiles, the accumulation of TrypPIs and peroxidase [4,8,31,32,33,34,35,36]. In this study, we found that bismerthiazol was not directly toxic to herbivores but enhanced levels of constitutive and herbivore-induced JA, JA-Ile, ET and H_2_O_2_, and these subsequently induced the resistance of rice to WBPH, BPH and SSB. Moreover, the exogenous application of bismerthiazol altered the volatile profile in rice and increased the attractiveness of plants to *A. nilarpavatae*, an egg parasitoid of rice planthoppers. The results demonstrate that bismerthiazol can act as a chemical elicitor to increase the resistance of rice to herbivores.

## 2. Results

### 2.1. Bismerthiazol Treatment Enhances the Direct Resistance of Rice to WBPH and Slightly Impairs Plant Growth

**Figure 1:**
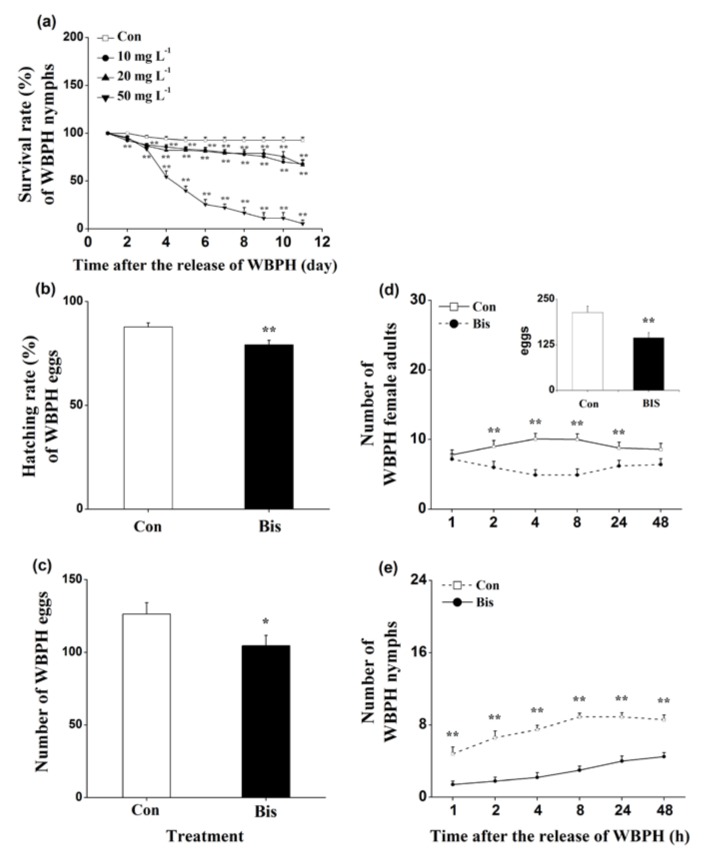
Bismerthiazol induces the direct resistance of rice to WBPH. (**a**) Mean survival rates (+ SE, *n* = 6) of 15 newly hatched WBPH nymphs fed on plants that had been grown in nutrient solution with either different concentrations of bismerthiazol (Bis) or without bismerthiazol (Con) for 24 h, 1–11 days after exposure; (**b**) Mean hatching rates (+SE, *n* = 10) of eggs laid over 12 h by 15 gravid WBPH females on plants that had been grown in nutrient solution with 50 mg L^−1^ bismerthiazol (Bis) or without bismerthiazol (Con) for 24 h; (**c**) Mean numbers (+SE, *n* = 10) of eggs laid over 12 h by 15 female WBPH adults fed on plants that had been grown in nutrient solution with 50 mg L^−1^ bismerthiazol (Bis) or without bismerthiazol (Con) for 24 h; (**d**) Mean number of gravid WBPH females (+SE, *n* = 10) fed on plants that had been grown in nutrient solution with 50 mg L^−1^ bismerthiazol (Bis) or without bismerthiazol (Con) for 24 h, 1–48 h after exposure. Inset shows the mean percentage (+SE, *n* = 10) of WBPH eggs per plant on pairs of plants as stated above, 48 h after the release of WBPH; (**e**) Mean number of newly hatched WBPH nymphs (+SE, *n* = 10) on plants that had been grown in nutrient solution with 50 mg L^−1^ bismerthiazol (Bis) or without bismerthiazol (Con) for 24 h, 1–48 h after exposure. For survival rate data, asterisks indicate significant differences in bismerthiazol-treated plants compared with non-treated plants (** *p* < 0.01; Duncan’s multiple-range test); for other data, asterisks indicate significant differences between members of a pair (* *p* < 0.05; ** *p* < 0.01; Student’s *t*-test).

**Figure 2:**
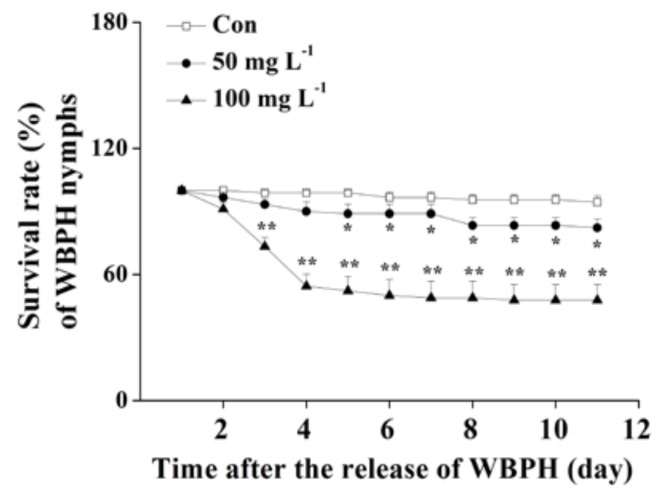
Spraying with bismerthiazol induces resistance of rice to WBPH. Mean survival rates (+SE, *n* = 6) of 15 newly hatched WBPH nymphs fed on plants that had been individually sprayed with 4 mL of 50 or 100 mg L^−1^ bismerthiazol (Bis) or with buffer only (Con) for 24 h, 1–11 days after exposure. Asterisks indicate significant differences in bismerthiazol-treated plants compared with non-treated plants (* *p* < 0.05; ** *p* < 0.01; Duncan’s multiple-range test).

**Figure 3:**
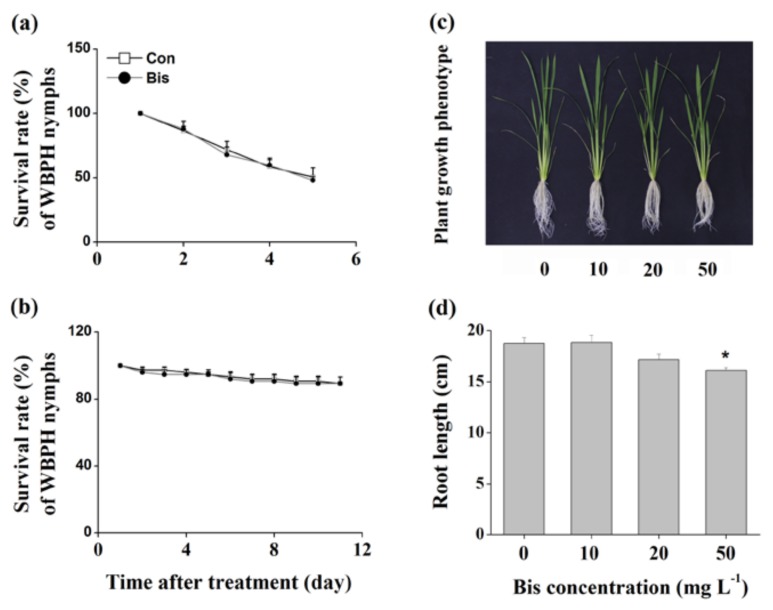
Bismerthiazol has no direct toxicity to WBPH but does slightly impair plant growth. (**a**) Mean survival rates (+SE, *n* = 6) of 15 newly hatched WBPH nymphs fed on artificial diets with 50 mg L^−1^ of bismerthiazol (Bis) or without bismerthiazol (Con); (**b**) Mean survival rates (+SE, *n* = 6) of 15 newly hatched WBPH nymphs that were exposed to 50 mg L^−1^ of bismerthiazol (Bis) or to buffer only (Con); (**c**) Growth phenotypes of plants grown in nutrient solutions with different concentrations of bismerthiazol for 10 days; (**d**) Mean root lengths (+ SE, *n* = 6) of plants grown in nutrient solutions with different concentrations of bismerthiazol for 10 days. Asterisks indicate significant differences between treatments and controls (* *p* < 0.05; Student’s *t*-test).

The survival rates of WBPH nymphs fed on plants that were grown in a nutrient solution with a concentration of 10, 20 or 50 mg L^−1^ bismerthiazol decreased by 26.9%, 28.1% and 94%, respectively, relative to untreated plants (Figure 1a). Moreover, lower hatching rates of WBPH eggs and smaller numbers of WBPH eggs laid by gravid females over 12 h on bismerthiazol-treated plants compared with rates and numbers on control plants were observed (Figure 1b,c). In a choice experiment, both gravid females and nymphs preferred to feed on control plants rather than on bismerthiazol-treated plants (Figure 1d,e); and gravid WBPH females laid more eggs on control plants than on bismerthiazol-treated plants (Figure 1d). Like plants whose roots had been treated with bismerthiazol, plants whose above-ground parts had been sprayed with the compound also had enhanced resistance to WBPH: the survival rate of WBPH nymphs fed on rice plants that had been individually sprayed with 4 mL of 50 or 100 mg L^−1^ bismerthiazol decreased by 12.94% and 49.41%, compared to the survival rate of nymphs fed on control plants (Figure 2). We found that the presence of bismerthiazol did not cause contact or stomach-poisoning toxicity in WBPH nymphs (Figure 3a,b). The data demonstrate that bismerthiazol probably affects the performance of WBPH via its induction of defense responses in rice.

We also examined the effect of bismerthiazol treatment on rice growth. Results showed that bismerthiazol has only a slight effect on rice growth: compared to plants growing in the nutrient solution without bismerthiazol, only 30-day-old plants growing in nutrient solution with a concentration of 50 mg L^−1^ bismerthiazol for 10 days exhibited slightly shorter roots (Figure 3c,d). No difference was observed in plant height, root mass and above-ground part mass between bismerthiazol-treated plants and control plants (Figure S1).

### 2.2. Bismerthiazol Induces the Biosynthesis of Constitutive and/or Elicited JA, JA-Ile, ET and H_2_O_2_, but Not SA

**Figure 4:**
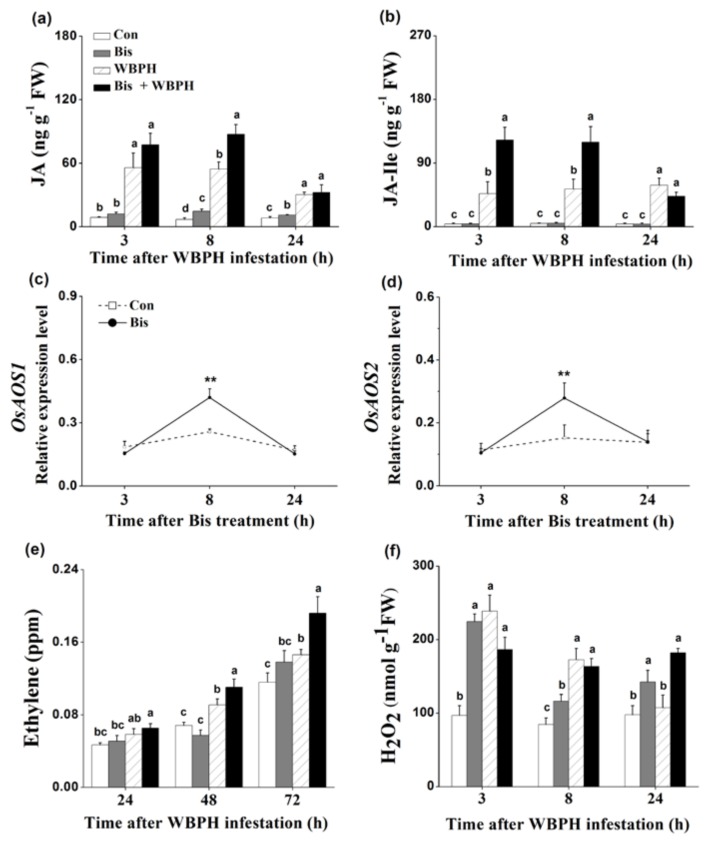
Bismerthiazol elicits JA, JA-Ile, ET and H_2_O_2_ biosynthesis. (**a**,**b**) Mean levels (+SE, *n* = 5) of JA (**a**) and JA-Ile (**b**) in leaf sheaths of rice plants that had been differently treated for 24 h, at 3, 8 and 24 h after being exposed to WBPH infestation. Con, control plants; Bis, bismerthiazol-treated plants; WBPH, WBPH-infested plants; Bis + WBPH, bismerthiazol + WBPH-treated plants. These treatment methods are described in Materials and Methods; (**c**,**d**) Mean relative expression levels (+SE, *n* = 5) of *OsAOS1* (**c**) and *OsAOS2* (**d**) in leaf sheaths of plants 3–24 h after plants were grown in nutrient solution with 50 mg L^−1^ bismerthiazol (Bis) or not (Con); (**e**) Mean levels (+SE, *n* = 10) of ethylene released from plants that had received different treatments for 24 h, at 24, 48 and 72 h after exposure to WBPH infestation. Treatments are as stated above; (**f**) Mean levels (+SE, *n* = 5) of H_2_O_2_ in leaf sheaths of plants that had received different treatments for 24 h, at 3, 8 and 24 h after exposure to WBPH infestation. Treatments are as stated above. Letters indicate significant differences between different treatments (*p* < 0.05, Duncan’s multiple-range test). Asterisks indicate significant differences between treatments and controls at each time point (** *p* < 0.01; Student’s *t*-test).

JA, JA-Ile, SA, ET and H_2_O_2_ are known to play a pivotal role in mediating plant defense responses [5,37,38]. Therefore, we examined whether bismerthiazol treatment induced the biosynthesis of these signaling molecules in plants. Since feeding on plants whose roots were treated with 50 mg L^−1^ bismerthiazol had the most lethal effect on WBPH nymphs, we used this concentration of bismerthiazol and root treatment to carry out experiments. Compared to non-infested plants, plants infested with gravid WBPH females displayed higher levels of JA, JA-Ile, ET and H_2_O_2_ (Figure 4), suggesting that exposure to infestation by gravid WBPH females could induce the production of all four of these signaling molecules. Bismerthiazol treatment alone significantly enhanced JA levels at 8 and 24 h after treatment, and the levels of JA in bismerthiazol-treated plants were 2.13-fold higher than those in control plants at 8 h (Figure 4a). Additionally, bismerthiazol treatment also enhanced JA levels in plants at 8 h after WBPH infestation (Figure 4a). Bismerthiazol treatment alone did not alter levels of JA-Ile; however, only when bismerthiazol-treated plants were exposed to infestation by gravid WBPH females did they display higher JA-Ile levels than those in control plants: JA-Ile levels in bismerthiazol-treated plants were approximately 2.63- and 2.25-fold higher than those in plants without bismerthiazol treatment at 3 and 8 h, respectively, following exposure to WBPH infestation (Figure 4b). Like the JA levels, the transcript levels of *OsAOS1* and *OsAOS2*, genes related to JA biosynthesis [39,40], increased at 8 h after treatment with bismerthiazol (Figure 4c,d). Bismerthiazol treatment only slightly enhanced SA levels in rice at 3 h after treatment and did not influence the production of SA in plants that had also been exposed to WBPH infestation plants (Figure S2).

As was observed in JA-Ile accumulation, bismerthiazol treatment alone did not induce the ET production in plants. Only when treated plants were exposed to WBPH infestation were higher ET levels in bismerthiazol-treated plants observed: levels from bismerthiazol-treated plants 48 and 72 h after exposure to infestation by WBPH were 121% and 132% of those in untreated plants that had been exposed to the infestation (Figure 4e). Bismerthiazol treatment alone enhanced H_2_O_2_ levels in plants: levels in plants treated with bismerthiazol at 3, 8 and 24 h, respectively, increased by 2.31-, 1.37- and 1.46-fold compared to those in control plants (Figure 4f). Twenty-four h after exposure to WBPH infestation, the H_2_O_2_ level in bismerthiazol-treated plants was 1.69-fold higher than the level in exposed plants without bismerthiazol treatment (Figure 4f). These findings suggest that bismerthiazol treatment was able to induce the production of constitutive and/or WBPH-elicited JA, JA-Ile, ET and H_2_O_2_ but not of SA.

### 2.3. Bismerthiazol Treatment Alters the Volatile Chemical Profile of Rice

As reported in previous results [8], non-manipulated rice plants released only a few volatile chemicals (Table 1). When treated with bismerthiazol, rice plants released more volatiles. The total amount of volatiles from bismerthiazol-treated plants was about 178% of the amount from control plants. Moreover, bismerthiazol treatment induced the production of 6 individual compounds—α-thujene, unknown 1, sesquithujene, (*E*)-α-bergamotene, α-curcumene and β-sesquiphellandrene—and increased levels of two chemicals, myrcene and methyl salicylate (MeSA) (Table 1, Figure S3). Exposure to infestation by gravid WBPH females drastically induced the production of volatiles in rice: the level of the total volatiles released from WBPH-infested plants was 14.6-fold higher than the level from non-infested plants. In addition, 11 chemicals that were not detected in non-infested plants—n-thujene, unknown 1, unknown 2, α-copaene, sesquithujene, (*E*)-α-bergamotene, sesquisabinene A, (*E*)-β-farnesene, α-curcumene, β-sesquiphellandrene and (*E*)-γ-bisabolene—were induced; and levels of 9 chemicals—2-heptanone, 2-heptanol, (+)-limonene, (*E*)-linalool oxide, linalool, MeSA, (*E*)-β-caryophyllene, zingiberene and β-bisabolene—were increased by exposure to WBPH infestation (Table 1). Interestingly, bismerthiazol treatment decreased the total amount of volatiles emitted from WBPH-infested plants, which was only 47.79% of the total amount of volatiles from plants exposed to WBPH infestation but not treatment. Furthermore, levels of 11 chemicals—2-heptanone, 2-heptanol, (*E*)-linalool oxide, linalool, unknown 1, sesquithujene, sesquisabinene A, zingiberene, β-bisabolene and β-sesquiphellandrene—in WBPH-infested plants were significantly decreased by bismerthiazol treatment (Table 1, Figure S3).

### 2.4. Bismerthiazol Treatment Enhances the Indirect Resistance of Rice to WBPH

**Figure 5:**
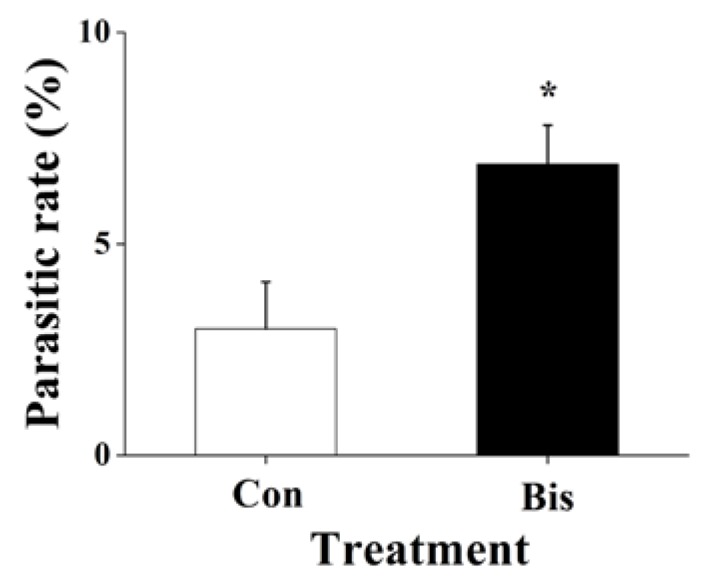
Mean parasitism rates (+ SE, *n* = 15) of WBPH eggs by *A. nilaparvatae* on the plants that had been grown for 1 day in nutrient solution with 50 mg L^−1^ bismerthiazol (Bis) or without bismerthiazol, 2 days after plants were placed into the field. Asterisks indicate significant differences between members of a pair (* *p* < 0.05; Student’s *t*-test).

Because bismerthiazol treatment changed the volatile profile of rice (Table 1), and plant volatiles play an important role in the host/prey-searching behavior of natural enemies of herbivores [41], we wanted to explore the effects of the bactericide on egg parasitism in the field. We found that bismerthiazol treatment affected the parasitism rate of WBPH eggs: eggs laid on bismerthiazol-treated plants by *A. nilaparvatae* were parasitized at rates 2.3-fold higher than eggs laid on plants without bismerthiazol treatment (Figure 5). This result indicates that bismerthiazol treatment was also able to increase the indirect resistance of rice to WBPH.

### 2.5. Bismerthiazol Treatment Also Enhances the Resistance of Rice to BPH and SSB

**Figure 6:**
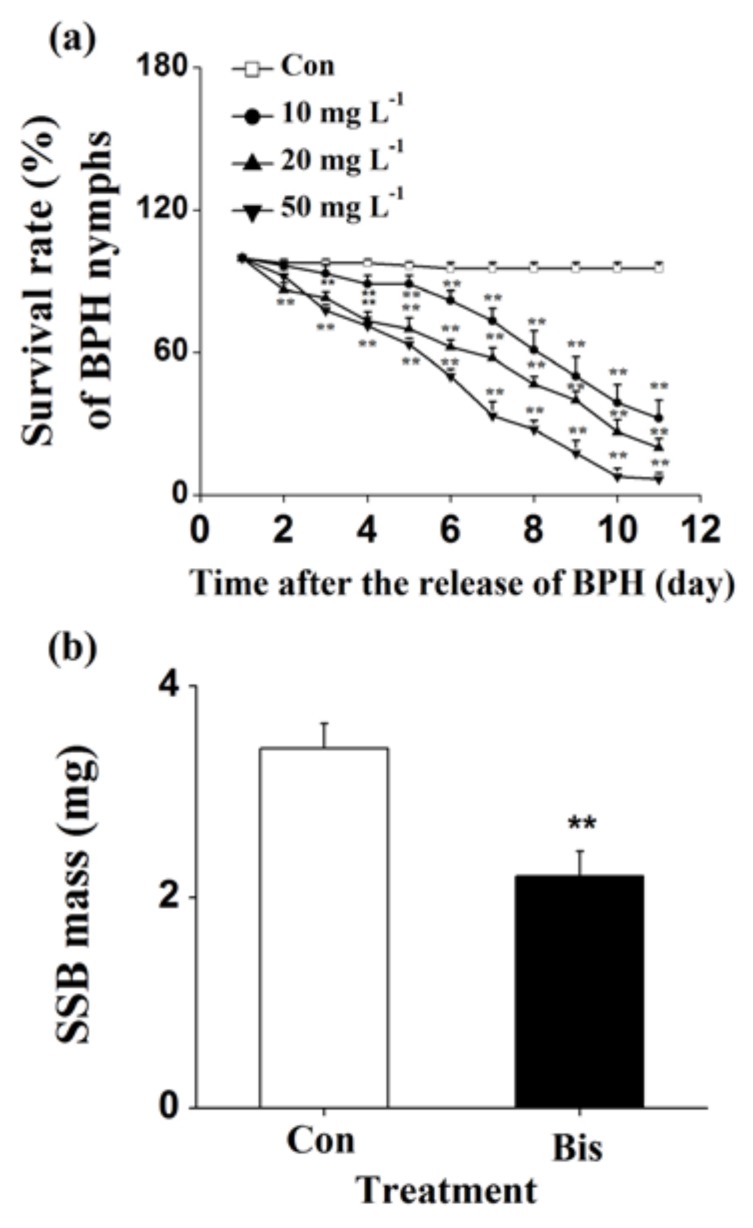
Bismerthiazol induces the resistance of rice to BPH and SSB. (**a**) Mean survival rates (+SE, *n* = 6) of 15 newly hatched BPH nymphs fed on plants that had been grown in nutrient solution with bismerthiazol (Bis) or without bismerthiazol (Con) for 24 h, 1–11 days after exposure; (**b**) Mean larval mass (+SE, *n* = 30) of SSB fed for 15 days on plants that had been grown in nutrient solution with 50 mg L^−1^ bismerthiazol (Bis) or without bismerthiazol (Con) for 24 h. For BPH data, asterisks indicate significant differences in bismerthiazol-treated plants compared with non-treated plants (** *p* < 0.01; Duncan’s multiple-range test); for SSB data, asterisks indicate significant differences between members of a pair (** *p* < 0.01; Student’s *t*-test).

To determine whether bismerthiazol induces the broad-spectrum resistance of rice to herbivores, we also measured the influence of bismerthiazol treatment on the performance of another piercing and sucking herbivore, BPH, and a chewing herbivore, SSB. The survival rate of BPH nymphs fed on plants that were grown in the nutrient solution with a concentration of 10, 20 or 50 mg L^−1^ bismerthiazol decreased by 66.3%, 79.1% and 93.0%, respectively, compared to the survival rate of BPH nymphs fed on plants that were grown in the control solution (Figure 6a). Similarly, the larval mass of SSBs fed on bismerthiazol-treated plants for 12 days decreased by 35.45% relative to the larval mass of those fed on untreated plants (Figure 6b).

## 3. Discussion

In the study, we discovered that bismerthiazol treatment both induced the biosynthesis of constitutive and/or WBPH-elicited JA, JA-Ile, ET and H_2_O_2_, and altered the volatile profile of rice (Figure 4 and Figure S3, Table 1). These changes enhanced the direct resistance of rice to WBPH, BPH and SSB, as well as the indirect resistance of rice to planthoppers, by attracting *A. nilaparvatae*, the egg parasitoid of rice planthoppers (Figure 1, Figure 2, Figure 5 and Figure 6). These findings demonstrate that bismerthiazol can act as a chemical elicitor that increases the resistance of rice to herbivores.

Signaling pathways mediated by JA, JA-Ile, ET, SA and H_2_O_2_ play a central role in regulating plant defense responses to herbivores [3,4,5,6]. Bismerthiazol has been reported to induce defense response in citrus by activating the SA signaling pathway but not the JA signaling pathway [42]. In rice, the exogenous application of bismerthiazol enhances H_2_O_2_ levels, up-regulates expression of defense-related genes, and induces both callose deposition and the hypersensitive response-like cell death in leaves infected with *Xoo* strain ZJ173 but not in non-infected leaves [29]. Here, we found that the exogenous application of bismerthiazol on rice induced the production of constitutive and/or elicited JA, JA-Ile, ET and H_2_O_2_ (Figure 4); however, the application had little or no effect on the production of SA (Figure S2). These discrepancies might be related to the different plant species and genotypes used. Further research should elucidate how biomerthiazol elicits these signaling pathways and how a plant’s genetic background influences induction.

In rice, both JA- and ET-mediated pathways positively regulate the plant’s resistance to chewing herbivores, such as SSB and leaf folder *Cnaphalocrocis medinalis*, by eliciting the accumulation of corresponding defensive compounds, such as TrypPIs [33,43,44]. In contrast, the JA-mediated pathway (probably by inhibiting the H_2_O_2_ pathway) and the ET-mediated pathway negatively modulate resistance of rice to piercing and sucking herbivores, such as BPH [8,33,34,45,46]; moreover, the H_2_O_2_-mediated pathway positively regulates the plant’s resistance to BPH [4,33,46,47,48]. Some researchers have shown that the activation of the SA pathway increases the resistance of rice to BPH [4,33,49]; however, it has been reported recently that the OsWRKY45-dependent SA pathway negatively modulates the resistance of rice to BPH [50]. Thus, higher elicited levels of H_2_O_2_ and of JA, JA-Ile and ET in bismerthiazol-treated plants compared to in untreated plants contributed at least partially to plant’s enhanced resistance to WBPH and BPH, as well as to SSB.

Volatile analysis found that the exogenous application of bismerthiazol on rice plants could induce the production of six volatile chemicals and enhance levels of two chemicals, although the amount of total volatiles was not much increased. In contrast, the exogenous application of bismerthiazol decreased production in plants exposed to WBPH infestation: levels of 11 chemicals were significantly decreased, and the amount of the total volatiles was only about half the amount in untreated plants that have exposed to WBPH infestation (Table 1). In rice, the exogenous application of JA induces the release of rice volatiles [51]; however, surprisingly, the ethylene-mediated pathway positively regulates the production of SSB-induced volatiles but negatively regulates the production of BPH-induced volatiles [44]. We found that bismerthiazol treatment induced the accumulation of JA, JA-Ile and ET in rice plants exposed to infestation by gravid WBPH females, whereas it induced only the biosynthesis of JA in non-infested plants (Figure 4). Thus, the way that the induction of biomerthiazol affects the production of volatiles in non-infested plants is probably related to how the induction regulates JA biosynthesis. The decrease in volatiles in bismerthiazol-treated plants that had been exposed to infestation compared to in exposed but untreated plants suggests that the ratio of JA to ethylene (or even the ratio of JA to other signals) is crucial for the production of rice volatiles. Interestingly, we found that bismerthiazol treatment decreased the level of MeSA in WBPH-infested plants but increased it in non-infested plants, compared to their corresponding control plants, although bismerthiazol treatment had little or no effect on SA levels in these plants (Figure S1). MeSA is produced via methylation of SA catalyzed by salicylic acid carboxyl methyltransferases (SAMTs) [52]. Therefore, these findings suggest that the effect of bismerthiazol treatment on the production of MeSA in rice plants might mainly be via its influence on the activity of SAMTs, while not on the biosynthesis of SA. Moreover, the data demonstrate that the activity of SAMTs is also affected by WBPH infestation and the interaction between bismerthiazol treatment and WBPH infestation. Further research should elucidate these issues.

Plant volatiles affect not only the host-searching behavior of insects but also their performance [32,53,54]. In addition to negatively affecting the performance of WBPH, bismerthiazol treatment also decreased the feeding and/or oviposition preference of gravid WBPH female adults and nymphs on plants (Figure 1d,e). Compared to plants without bismerthiazol treatment, treated plants released more volatiles, including 11 chemicals that were not detected in untreated plants and 2 chemicals whose levels were increased (Table 1). Moreover, one of these chemicals, methyl salicylate, was found to repel BPH [55]. Thus, apart from the other defenses mediated by enhanced signaling pathways, the change of volatiles in bismerthiazol-treated plants also partially contributed to the enhanced direct resistance to herbivores, reducing the performance and altering the preference of herbivores.

Interestingly, unlike the result that WBPH nymphs and gravid females did not prefer to feed and/or lay eggs on bismerthiazol-treated plants, the parasitoid *A. nilaparvatae* did prefer to parasitize WBPH eggs laid on bismerthiazol-treated plants over eggs laid on untreated plants: in the field, the parasitism of WBPH eggs by *A. nilaparvatae* on treated plants was 2.3-fold higher than the parasitism on untreated plants (Figure 5). Plant growth phenotype can also influence the host-searching behavior of parasitoids [56]. However, we could exclude the effect of plant growth phenotype on the parasitism rate of WBPH eggs as there was only a small difference in the growth phenotypes of bismerthiazol-treated and non-treated plants even 10 days after treatment (Figure 3c,d), and in this experiment, we treated plants only for 1 day. Thus, the observed difference in the amount of parasitism of WBPH eggs between bismerthiazol-treated and untreated plants is probably due mainly to the difference in volatile amounts between the two groups of plants. Surprisingly, bismerthiazol treatment decreased the production of all detected volatiles from WBPH-infested plants, including the three chemicals attractive to the parasitoid: linalool, MeSA and (*E*)-β-caryophyllene [41,55,57] (Table 1). Similarly, in maize, treatment with BTH or laminarin is reported to reduce herbivore-induced volatile emissions but increase the plant’s attractiveness to parasitoids [24]. These findings demonstrate that the ratio of volatile chemicals or as-yet undetectable and unidentified chemicals may be of great importance for attracting *A. nilaparvatae*. Another possibility is that the volatiles that negatively affect *A. nilaparvatae* may also be the ones that are reduced. The exact mechanisms underlying this biological phenomenon should be clarified in future research.

In summary, our results demonstrate that bismerthiazol can enhance constitutive, especially herbivore-elicited, levels of defense-related signals, such as JA, JA-Ile, ET and H_2_O_2_. These activated signaling pathways alter the chemical profile of plants, and the changes, in turn, increase both direct and indirect resistance of rice to herbivores. As a commonly used bactericide, bismerthiazol is generally used to control bacterial diseases in rice and its amount applied in the field is about 375 g active ingredient per hectare [58]. The number of rice plants is about 2.5–3.0 million per hectare. Thus, the amount of bismerthiazol received by one plant (about 0.25–0.30 mL of 500 mg L^−1^ bismerthiazol per plant) in the field is comparable to the amount received by one plant that was sprayed with 4 mL of 100 mg L^−1^ bismerthiazol in this study (we suppose that about half of the amount of bismerthiazol used reached the plant). This indicates that bismerthiazol has a potential to be used in the field as a chemical elicitor for insect pest management. Further researches should investigate the control effect of bismerthiazol on rice insect pests in the field. Moreover, given that bismerthiazol is an efficient bactericide and that bismerthiazol can induce plant defenses against pathogens [29,59], these findings also open a pathway for controlling diseases and insect pests by designing and exploiting chemical elicitors that have such characteristic structures.

## 4. Materials and Methods

### 4.1. Plant Material and Growth Conditions

The rice (*Oryza sativa*) genotypes used in this study was Xiushui 110. Seeds were germinated in water for 1 day and cultured in plastic bottles (diameter 8 cm, height 10 cm) in a greenhouse (28 ± 2 °C, 14 h light, 10 h dark). Ten-day-old seedlings were transferred to 20 L communal hydroponic boxes with rice nutrient solution [60]. After 30–35 days, seedlings were transferred to individual 320 mL hydroponic plastic pots. Rice plants were used for experiments 3 days after transplanting.

### 4.2. Compounds

Bismerthiazol (purity, 98%) was provided by Longwan Agrichemical Co. Ltd., Wenzhou, China. *N*,*N*-Dimethylformamide and Tween-60 were purchased from Sinopharm Chemical Reagent Co. Ltd., Shanghai, China.

### 4.3. Insects

Colonies of WBPH, BPH and SSB were originally obtained from rice fields in Hangzhou, China, and maintained on TN 1 (a variety susceptible to the three herbivore species) rice seedlings in a controlled climate room (26 ± 2 °C, 14 h light, 10 h dark, 80% relative humidity).

### 4.4. Plant Treatments

For bimerthiazol treatment, two methods were used: root treatment and spray treatment. For root treatment, plants were grown in nutrient solution, and bimerthiazol (first dissolved in *N*,*N*-dimethylformamide, 1 mg per mL of *N*,*N*-dimethylformamide)—at final concentrations ranging from 10 to 50 mg L^−1^—was added (with 0.01% Tween-60). Control plants (Con) were grown in nutrient solution without bismerthiazol but with an equal volume of *N*,*N*-dimethylformamide and Tween-60. For spray treatment, bismerthiazol was first dissolved in *N*,*N*-dimethylformamide (1 mg per mL of *N*,*N*-dimethylformamide) and then diluted in distilled water (with 0.01% Tween-60) at concentrations ranging from 50 to 100 mg L^−1^. The above-ground parts of each plant were sprayed using an atomizer with 2 mL of bismerthiazol solution twice at interval of 4 h. Control plants were sprayed with distilled water containing the same volume of *N*,*N*-dimethylformamide and Tween-60. For WBPH treatment, individual plants were infested with 15 WBPH nymphs or gravid females that were confined within a glass cylinder (diameter 4 cm, height 8 cm, with 48 small holes, diameter 0.8 mm), and the top of the cylinder was covered with a piece of sponge. Empty cylinders were attached to control plants (non-infested plants).

### 4.5. Measurement of Plant Growth Parameters

Plant growth parameters, including plant height, root length, and mass of above- and below-ground parts of control plants and bismerthiazol-treated plants (grown in nutrient solution with different concentrations of bismerthiazol) were measured at 10 days after bismerthiazol treatment. Plant height and root length were defined as the part of a plant from the stem base to the longest leaf apex and that from the stem base to the longest root tip, respectively. Plants were cut off from the stem base and then the mass of above-ground and below-ground (roots) part of plants was measured. The experiment was replicated 6 times.

### 4.6. qRT-PCR

For qRT-PCR analysis, five independent biological samples were used. Total RNA was isolated using the SV Total RNA Isolation System (Promega, Madison, WI, USA) following the manufacturer’s instructions. One microgram of each total RNA sample was reverse-transcribed using the PrimeScript RT-PCR Kit (TaKaRa, Shiga, Japan). The QRT-PCR assay was performed on a CFX96TM Real-Time system (Bio-Rad, Hercules, CA, USA) using a Premix Ex TaqTM Kit (TaKaRa, Shiga, Japan). A linear standard curve, threshold cycle number versus log (designated transcript level), was built using a series concentrations of a specific cDNA standard. Relative levels of the transcript of the target gene in tested samples were calculated according to the standard curve. A rice actin gene *OsACT* (TIGR ID: Os03g50885) was used as an internal standard to normalize cDNA concentrations. The primers and probes used for qRT-PCR for all tested genes are listed in Table S1.

### 4.7. WBPH and BPH Performance Measurement

The survival rates of WBPH and BPH nymphs on bismerthiazol-treated plants and control plants were investigated. For WBPH bioassays, two bismerthiazol treatments, root and spray, were used. Twenty-four hours after plants had been treated with bismerthiazol, the basal stem of each plant was covered in a glass cylinder into which 15 newly hatched WBPH or BPH nymphs were introduced. Every day, the number of surviving WBPH or BPH nymphs and emerging adults on each plant was recorded until all the nymphs had become adults. The experiment was repeated 6–10 times.

The hatching rates of WBPH eggs on bismerthiazol-treated plants (plants that had been grown in nutrient solution containing bismerthiazol for 24 h) and control plants were determined. Fifteen gravid WBPH females were exposed to individual plants for 12 h, and then all the insects were removed. The number of freshly hatched WBPH nymphs on plants was recorded every day until no new nymphs occurred for three consecutive days. Unhatched eggs and total eggs were counted to determine the hatching rate on each plant. The experiment was repeated 6 times.

To determine the effect of bismerthiazol treatment on the colonization and/or oviposition preferences of gravid WBPH females or nymphs, two plants (one control plant and one bismerthiazol-treated plant, as stated above) were confined within a glass cylinder into which 15 gravid females or nymphs were introduced. The number of gravid WBPH females or nymphs on each plant was counted 1, 2, 3, 8, 24 and 48 h after the release of WBPH; after 48 h, WBPH were removed and the eggs on each plant (for experiments with gravid females) were counted. The experiment was repeated 10 times.

To detect the direct effect of bismerthiazol on the survival rate of newly hatched WBPH nymphs, the contact toxicity and the stomach-poisoning toxicity of bismerthiazol were measured. For the contact toxicity measurement, 15 newly hatched WBPH nymphs were placed into a Petri dish (diameter 9 cm, height 2 cm) lined with filter paper wetted with 0.2 mL of 50 mg L^−1^ bismerthiazol. Controls were lined with filter paper wetted with 0.2 mL of distilled water. After 2 h, WBPH nymphs were transferred onto the basal stem of each plant and covered in a glass cylinder. The experiment was repeated 6 times. For stomach-poisoning toxicity measurements, 15 newly hatched WBPH nymphs were fed on artificial diet with a concentration of 50 mg L^−1^ bismerthiazol in a 30 mL glass cylinder with two ends open (diameter 9 cm, height 2 cm) as described in [61]. Controls were fed on artificial diet without bismerthiazol. Each treatment was replicated 5 times. Every day, the number of surviving WBPH nymphs and emerging adults on each plant in both experiments was recorded until all the nymphs had become adults.

### 4.8. SSB Performance Measurement

To measure the effect of bismerthiazol treatment on the performance of SSB, individual bismerthiazol-treated plants (as stated in Section 4.6) or control plants were infested with a newly hatched SSB larva. Larval mass was measured 15 days after the release of the herbivore. The experiment was repeated 30 times.

### 4.9. JA, JA-Ile, SA, ET and H_2_O_2_ Analysis

For JA, JA-Ile, and SA analysis, both bismerthiazol-treated plants (as stated in Section 4.6) and control plants were individually infested by 15 gravid WBPH females. Plant leaf sheaths were harvested at 3, 8 and 24 h after the start of WBPH infestation. Samples were ground in liquid nitrogen, and JA and JA-Ile were extracted with ethyl acetate spiked with labeled internal standards (^2^D2-JA, ^2^D6-JA-Ile and ^2^D4-SA) and analyzed with HPLC/mass spectrometry/mass spectrometry following the method described [36]. Each treatment at each time interval was replicated five times.

For ET analysis, bismerthiazol-treated plants (as stated in Section 4.6) and control plants were individually infested by 15 gravid WBPH females. Plants were then individually covered with sealed glass cylinders (diameter 4 cm, height 50 cm). ET production was determined at 24, 48 and 72 h after the start of WBPH infestation by using the method described previously [62]. Each treatment at each time interval was replicated 10 times.

For H_2_O_2_ analysis, both bismerthiazol-treated plants (as stated in Section 4.6) and control plants were individually infested by 15 gravid WBPH females. Plant leaf sheaths were harvested at 3, 8 and 24 h after WBPH infestation. The H_2_O_2_ concentrations were determined by using the Amplex^®^ Red Hydrogen Peroxide/Peroxidase Assay Kit (Invitrogen, available online: http://www.invitrogen.com/) as described previously [4]. Each treatment at each time interval was replicated five times.

### 4.10. Collection, Isolation and Identification of Volatile Compounds

The collection, isolation and identification of rice volatiles were carried out using the same method as our previously described [32,63]. Volatiles emitted from individual plants with different treatments—bismerthiazol-treated plants, WBPH-infested plants, bismerthiazol + WBPH-treated plants and control plants—were collected and lasted for 8 h. For control plants, plants were grown in the control solution for 1 day, followed by non-infestation for 12 h. For the WBPH treatment, plants were grown in the control solution for 1 day, followed by infestation by 15 gravid WBPH females for 12 h. For bismerthiazol-treated plants, plants were grown in nutrient solution with 50 mg L^−1^ bismerthiazol for 1 day, followed by non-infestation for 12 h. For bismerthiazol + WBPH-treated plants, plants were grown in nutrient solution with 50 mg L^−1^ bismerthiazol for 1 day, followed by infestation by 15 gravid WBPH females for 12 h. Each treatment was replicated five times. The detected compounds were expressed as the percentage of peak areas relative to the internal standard (IS, diethyl sebacate) per 8 h of trapping for one plant.

### 4.11. Field Experiment

To evaluate the effect of bismerthiazol treatment on the host-searching behavior of the parasitoid *A. nilaparvatae* in the field, experiments were carried out in November 2016 in a rice field in Lin’an, Hangzhou, China. Gravid WBPH females laid eggs on both bismerthiazol-treated plants (as stated in Section 4.6) and control plants for 24 h, and plants were then transferred to fields. Pairs of potted plants (one bismerthiazol-treated plant and one control plant), 20 cm apart each other, were placed at 15 locations (3 m apart) parallel to the ridge (1 m from the ridge) in a rice field. Plants were collected 2 days after plants were introduced into the field. The plants were transferred to the controlled climate room (28 ± 2 °C, 14 h light, 10 h dark, 80% relative humidity), and each pot was confined in a plastic cage (diameter 11 cm, height 10 cm) (herbivores, predators, and parasitoids on plants were all removed). Five days later, the plants were cut off at the soil level and dissected under a microscope to record the total number of WBPH eggs and the number of parasitized eggs.

## Figures and Tables

**Table 1:** Comparison of volatile compounds (mean ± SE, *n* = 6) emitted from control plants, bismerthiazol-treated plants, WBPH-infested plants and bismerthiazol + WBPH plants. These treatment methods were described in the section of Materials and Methods.

No	Compound	Con	Bis	WBPH	Bis + WBPH
1	2-Heptanone	4.14 ± 1.59 ^b^	5.70 ± 1.69 ^b^	15.77 ± 1.23 ^a^	6.64 ± 0.85 ^b^
2	2-Heptanol	0.49 ± 0.22 ^b^	0.50 ± 0.15 ^b^	4.13 ± 0.44 ^a^	0.59 ± 0.16 ^b^
3	α-Thujene	-	0.29 ± 0.17 ^a^	0.68 ± 0.17 ^a^	0.44 ± 0.04 ^a^
4	α-Pinene	0.53 ± 0.32 ^a^	0.94 ± 0.43 ^a^	0.81 ± 0.23 ^a^	0.71 ± 0.17 ^a^
5	Myrcene	1.20 ± 0.26 ^b^	2.12 ± 0.26 ^a^	1.72 ± 0.33 ^ab^	1.19 ± 0.26 ^b^
6	(+)-Limonene	0.86 ± 0.19 ^b^	1.69 ± 0.51 ^b^	11.95 ± 3.02 ^a^	10.70 ± 0.89 ^a^
7	(*E*)-Linalool oxide	0.14 ± 0.03 ^c^	0.32 ± 0.09 ^c^	2.98 ± 0.62 ^a^	0.93 ± 0.23 ^b^
8	Linalool	0.56 ± 0.07 ^c^	0.57 ± 0.23 ^c^	63.74 ± 15.76 ^a^	26.91 ± 4.48 ^b^
9	Methyl salicylate	0.15 ± 0.02 ^d^	0.41 ± 0.16 ^c^	6.05 ± 1.0 4 ^a^	2.06 ± 0.45 ^b^
10	Unknown 1	-	1.02 ± 0.28 ^a^	1.34 ± 0.26 ^a^	0.54 ± 0.10 ^b^
11	Unknown 2	-	-	1.28 ± 0.41 ^a^	0.33 ± 0.16 ^a^
12	α-Copaene	-	-	1.15 ± 0.37 ^a^	1.24 ± 0.04 ^a^
13	Sesquithujene	-	0.37 ± 0.10 ^b^	1.05 ± 0.21 ^a^	0.50 ± 0.02 ^b^
14	α-Cedrene	0.32 ± 0.04 ^a^	0.59 ± 0.14 ^a^	0.75 ± 0.16 ^a^	0.55 ± 0.06 ^a^
15	(*E*)-β-Caryophyllene	0.65 ± 0.07 ^b^	1.11 ± 0.39 ^b^	2.41 ± 0.46 ^a^	1.67 ± 0.14 ^ab^
16	(*E*)-α-Bergamotene	-	0.20 ± 0.08 ^b^	3.35 ± 0.92 ^a^	1.67 ± 0.10 ^a^
17	Sesquisabinene A	-	-	3.30 ± 0.87 ^a^	1.71 ± 0.12 ^b^
18	(*E*)-β-Farnesene	-	-	2.91 ± 0.83 ^a^	1.30 ± 0.10 ^a^
19	α-Curcumene	-	0.94 ± 0.34 ^a^	2.79 ± 0.81 ^a^	1.33 ± 0.16 ^a^
20	Zingiberene	1.13 ± 0.27 ^c^	1.41 ± 0.27 ^c^	10.33 ± 2.74 ^a^	5.08 ± 0.48 ^b^
21	β-Bisabolene	0.40 ± 0.11 ^c^	-	4.51 ± 1.27 ^a^	1.95 ± 0.20 ^b^
22	β-Sesquiphellandrene	-	0.48 ± 0.07 ^c^	7.70 ± 2.19 ^a^	3.72 ± 0.34 ^b^
23	(*E*)-γ-Bisabolene	-	-	3.78 ± 1.09 ^a^	2.09 ± 0.25 ^a^
Total		10.57 ± 3.17 ^c^	18.80 ± 5.36 ^c^	154.48 ± 35.44 ^b^	73.83 ± 9.78 ^b^

Letters in the same row indicate significant differences between treatments (*p* < 0.05, Duncan’s multiple-range test).

## Supplementary Materials

Supplementary materials can be found at http://www.mdpi.com/1422-0067/19/5/1271/s1. Table S1: Primers and probes used for QRT-PCR of target genes; Figure S1: Growth phenotypes of bismerthiazol-treated and control rice plants; Figure S2: Mean levels (+ SE, *n* = 5) of SA in leaf sheaths of rice plants with different treatments; Figure S3: Typical chromatograms obtained by head space collections from plants with different treatments.

## Abbreviations

JA	Jasmonic acid
JA-Ile	Jasmonoyl-isoleucine conjugate
SA	Salicylic acid
ET	Ethylene
BTH	Benzo (1,2,3) thiadiazole-7-carbothioic acid *S*-methyl ester
MeJA	Methyl ester of JA
2,4-D	2,4-dichlorophenoxyacetic acid
TrypPIs	Trypsin proteinase inhibitors
SSB	Striped stem borer
BPH	Brown planthopper
*Xoo*	*Xanthomonas oryzae pv. Oryzae*
WBPH	white-backed planthopper
MeSA	Methyl salicylate
SAMT	Salicylic acid carboxyl methyltransferase
