# Supplementary material for: The Commonly Used Bactericide Bismerthiazol Promotes Rice Defenses against Herbivores
Source: Int J Mol Sci. 2018 Apr 24;19(5):1271. doi: 10.3390/ijms19051271 (PMC5983687; doi:10.3390/ijms19051271)
Supplement: Supplementary file 1 [file ijms-19-01271-s001.pdf]

Supplementary Materials: The Commonly Used Bactericide Bismerthiazol Promotes Rice Defenses against Herbivores

Pengyong Zhou, Xiaochang Mo, Wanwan Wang, Xia Cheng and Yonggen Lou

Table S1. Primers and probes used for QRT-PCR of target genes.

Gene	RGAP ID	Primer (5′-3′)	Probe (5′-3′)
OsACT	Os03g50885	F: TGGACAGGTTATCACCATTGGT<br>R: CCGCAGCTTCCATTCCTATG	CGTTTCCGCTGCCCTGAGGTCC
OsAOS1	Os03g55800	F: CGAGCTCTTCCTCCGATACG<br>R: GTCAGAAGGTGGCCTTCTTGAG	ACCTCCACGCTCGGCTCATCTGTC
OsAOS2	Os03g12500	F: GCGCACGGGCTATTTC<br>R: CGATCACCGTTCACGATGAA	CGAGTAGTTAGGCGTCCAAAGTTTCGGGA

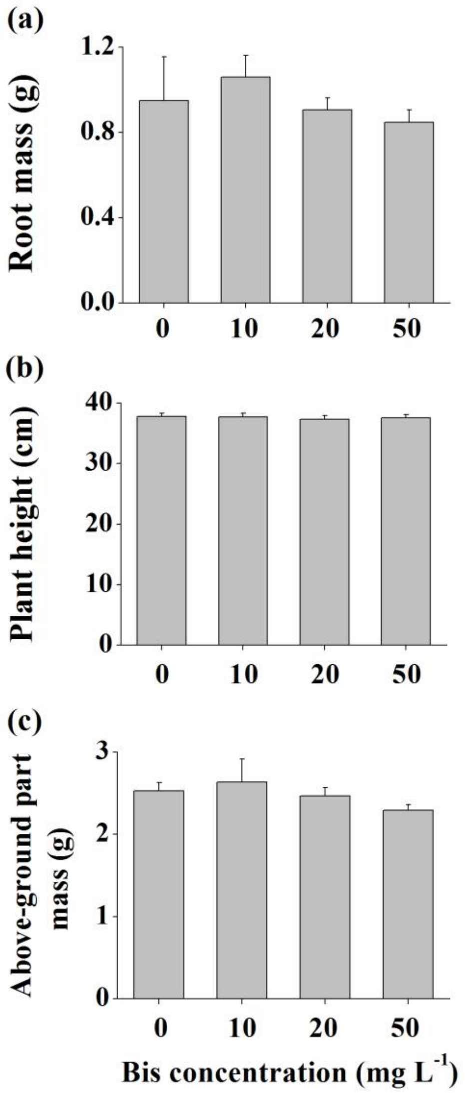

Figure S1. Growth phenotypes of bismerthiazol-treated and control rice plants. Mean root mass (**a**), plant height (**b**) and above-ground part mass (**c**) (+ SE, n = 6) of rice plants that had been grown in nutrient solution with different concentrations of bismerthiazol for 10 days.

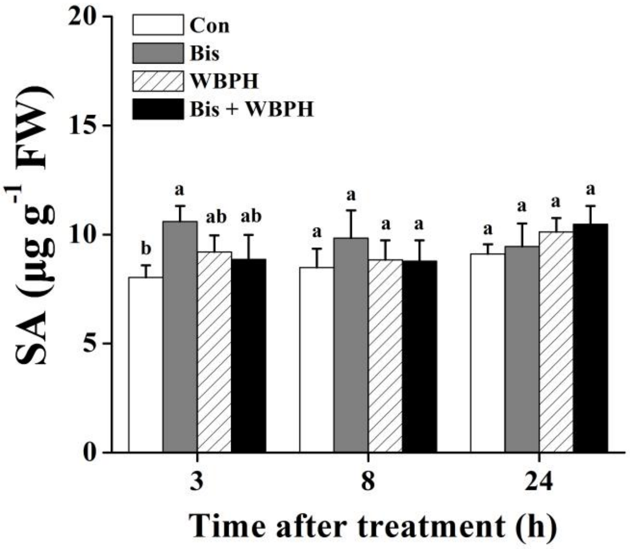

Figure S2. Mean levels (+ SE, *n* = 5) of SA in leaf sheaths of rice plants with different treatments. Con, control plants; Bis, bismerthiazol-treated plants; WBPH, WBPH-infested plants; Bis + WBPH, bismerthiazol + WBPH-treated plants. These treatment methods are described in Materials and Methods. Letters indicate significant differences between different treatments ($p < 0.05$, Duncan's multiple-range test).

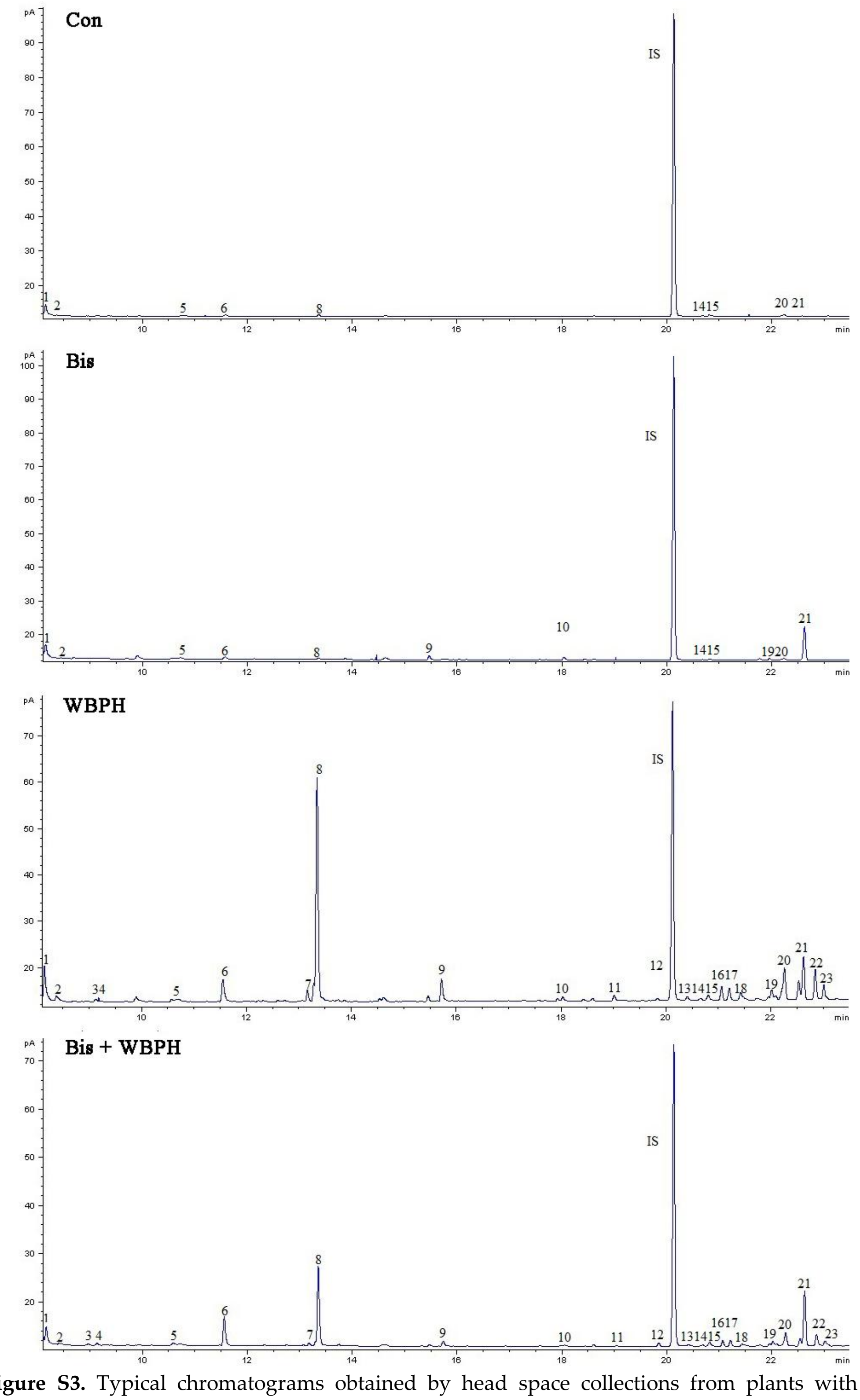

Figure S3. Typical chromatograms obtained by head space collections from plants with different treatments. Con, control plants; Bis, bismerthiazol-treated plants; WBPH, WBPH-infested plants; Bis + WBPH, bismerthiazol + WBPH-treated plants. These treatment methods are described in Materials and Methods. Numbers represent chemicals that are the same as in Table 1.
